# In situ monitoring of thin alumina passive film growth by surface plasmon resonance (SPR) during an electrochemical process

**DOI:** 10.1038/s41598-024-64378-w

**Published:** 2024-06-14

**Authors:** J. Dutems, N. Crespo-Monteiro, F. Faverjon, V. Gâté, D. Turover, S. Marcellin, B. Ter-Ovanessian, C. Héau, I. Verrier, B. Normand, Y. Jourlin

**Affiliations:** 1 CNRS, Institut d’Optique Graduate School, Laboratoire Hubert Curien UMR 5516, Université Jean Monnet Saint-Etienne, 42023 Saint-Étienne, France; 2 HEF IREIS, 42160 Andrézieux-Bouthéon, France; 3 SILSEF, 74160 Archamps, France; 4 CNRS, MATEIS, UMR5510, Univ Lyon, INSA Lyon, Université Claude Bernard Lyon 1, 69621 Villeurbanne, France

**Keywords:** Techniques and instrumentation, Applied optics

## Abstract

This article presents a sensing technique to characterize the growth of an alumina passive film on an aluminum micro structured layer in situ. The technique uses surface plasmon resonance (SPR) on aluminum coated gratings with spectroscopic measurements during electrochemical polarization in 0.02M Na_2_SO_4_. The structure of the sensor was first simulated and then fabricated by photolithography. The grating was then replicated by nanoimprint (NIL) in Sol–Gel before pure aluminum layer was deposited by RF magnetron sputtering to produce the samples used in this study. Coupled plasmonic and electrochemical measurements confirmed the feasibility of in situ characterization (thickness) of alumina passive film on aluminum-based gratings in neutral aqueous media. Combining both measurements with an appropriated SPR spectrum fitting lead to alumina thickness monitoring within a few nanometers’ accuracy. The objectives and challenges of this study are to better characterize the alumina growth during electrochemical process combining in situ electrochemical process and SPR spectra in order to determine thin passive layer characteristics.

## Introduction

Surface plasmon resonance (SPR) is a widely used sensing technique to detect small variations in the refractive index at the metal/dielectric interface. Functionalizing the metal surface makes it possible to use SPR as sensors^1^ such as biosensors to detect proteins^2^ or bacteria^3^ as well as to detect gases in the environment^4^. SPR is also exploited to characterize thin metal film, in order to measure dielectric properties^5^ or thin films thickness^6^.

Recently SPR was used to detect condensation phenomenon at the interface of acetone vapor and aluminum based diffraction grating^7^. In this study the authors exploited surface plasmon polaritons (SPP), which are considered as non-localized surface plasmons. Thus, surface plasmons polaritons are surface waves propagating along a metal/dielectric interface which occurs only for a specific wavelength (SPR resonance wavelength), angle and polarization (TM or p-polarized) incident wave. The electrical field of this SP mode is evanescent in the metal and the dielectric media with different penetration depths. Accordingly, SPP is well adapted to monitor interface modification. It is why the authors exploited SPP for the characterization of the alumina growth at the metal interface.

To enable the existence of the surface plasmon, phase matching must occur between the incident TM polarized light and the propagation constant of the plasmon at the metal/dielectric interface. Phase matching is achieved by exciting the plasmon mode with a prism in the Kretschmann-Raether and Otto configurations, or with a diffraction grating^1^. In the Kretschmann-Raether configuration, the metal layer used is extremely thin in order to be optically transparent, and the dielectric layer is considered as semi-infinite. In the Otto configuration, the dielectric layer is extremely thin to allow plasmonic coupling with a bulk metal layer. While in the diffraction grating configuration, the metal layer is generally considered to be semi-infinite. The choice of the coupling configuration will depend on the targeted application.

A link was established between a variation in the potential applied to a gold working electrode and the modification of the plasmonic angular dip with the Kretschmann-Raether configuration^8^. This modification was attributed to a variation in the double layer capacitance of the thin oxide film. Other authors^9^ studied and compared the growth of a gold oxide layer in H_2_SO_4_ and HClO_4_ solution with an ellipsometry setup enhanced with surface plasmon. Jory et al.^10^ implemented a device to study the gold/sulfuric acid interface with SPR using grating in order to correlate the plasmonic response with the charge zone of the electrochemical double layer with an applied potential for biosensor application. They concluded that the grating configuration makes it possible to obtain more robust electrodes for coupled plasmonic and electrochemical measurements, plus to overcome the problems encountered with both the Otto and Kretschmann-Raether configurations. Kosako et al.^11^ focused on detecting the initial stage of aluminum pitting corrosion in 3 wt% NaCl solution using SPR with the Kretschmann-Raether configuration. These authors investigated modifications in the thickness of the aluminum layer consumed during exposure of the metal with chloride ions without any applied potential. They were able to correlate the shift in experimental resonance of this consumption by simulating the thinning of the aluminum layer using different fixed passive film thicknesses, and concluded that the resonance shift they observed was caused by consumption of the aluminum layer. In the literature, it exists previous studies for the characterization of oxide films, especially using ellipsometry based measurements^12,13^. They are different from our approach because there are less sensitive for very thin layers (below 10 nm) and very accurate for higher thickness, contrary to SPP analysis which is all the more sensitive for low thicknesses, due to the high field enhancement at the interface metal /electrolyte. We consider that these two approaches are complementary to characterize thin passive films from a few nm to several hundreds of nm.

A passive film is a complex oxide layer formed on the surface of a passive metal, for example, aluminum or stainless steel. This film can protect the metal against corrosion. Understanding and controlling this phenomenon is of interest to both the scientific^14,15^ and industry^16,17^ communities. Since the complexity and metastable properties of the phenomenon are intrinsically linked to the environmental medium of the metal, it makes them very difficult to characterize. For a better understanding of their behavior during the oxide film growth, we need to increase our knowledge on their physicochemical properties. More precisely, we need to quantify the physical properties, like the thickness of a passive film, during the metastable phase to be able to modelize the passive film.

Electrochemical measurements and physicochemical techniques are usually used to characterize passive films. Measuring their polarization enables identification of the main electrochemical processes that take place in the anodic and cathodic domains, such as their corrosion potential. However, complementary measurements are needed to obtain information concerning the transport and the resistivity of the passive film or on its interfacial capacitance of the film^18^. Several surface techniques are also used to study passive films. The most common is the X-ray photoelectron spectroscopy (XPS)^19,20^. XPS measurements enable quantification of the thickness of thin oxide film^21,22^ as well as identification of the chemical composition of the surface^23^. Since this method is based on fitting results, the results require interpretation that is not always accurate.

The present work focuses on the feasibility of in situ detection of the growth of an alumina passive film induced by an applied potential in an electrochemical set-up combined with a real time recorded SPR spectra. Alumina has been chosen for its well-controlled growth on aluminum layer which is considered as an interesting and well known noble plasmonic metal working in the visible range even though it is more lossy compared to gold or silver layer^24^.

For better understanding passive films, we tested if a device that uses surface plasmon resonance is able to detect the growth of an aluminum passive film induced by an applied potential in situ. Next, we describe how we implement such experimental set-up with a micro structured aluminum-based substrate using photolithography and nanoimprinting, which enabled both spectroscopic and electrochemical measurements. Finally, we present the results of our data analysis and compared both measurements.

## Results and discussion

### Diffraction grating parameters

The sample used in this study was composed of an aluminum diffraction sinusoidal grating immersed in electrolyte. Sinusoidal gratings have been shown to have better plasmonic sensitivity than rectangular structures. The sinusoidal configuration makes it possible to obtain a greater plasmonic shift with the same variation in the refractive index at the metal/dielectric interface^25^.

**Figure 1:**
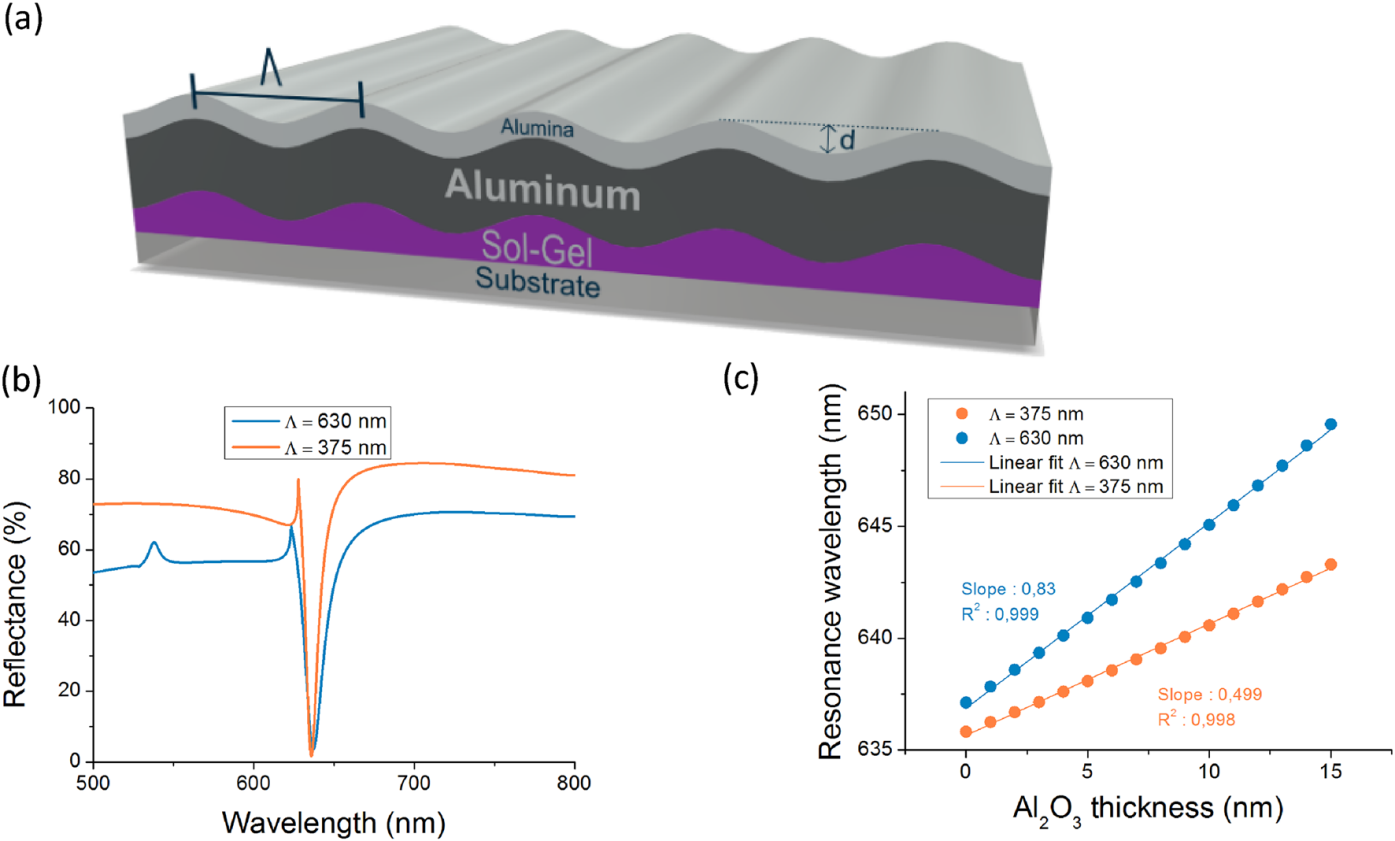
(**a**) Structure of the simulated diffraction grating with period Λ and the grating depth d. (**b**) Reflectance response as a function of wavelength under the simulated configuration. In orange, Λ = 375 nm with grating depth d = 36 nm. In blue, Λ = 630 nm with grating depth d = 54 nm. (**c**) Simulation of the growth af an Al_2_O_3_ for both structures.

The structure of the simulated figure is shown in Fig. 1a. The grating parameters should make it possible to obtain a plasmonic dip of the 0th reflected order; in the visible spectrum at wavelength λ = 635 nm for a fixed incident angle of 20° in air. The dip should have a spectral shift during the aluminum oxide growth while keeping a thin full width at half maximum (FWHM). For the modeling, permittivity of both alumina and aluminum layers has been extracted from Palik handbook^26^ whose values are implemented in the modeling code.

The first simulated structure had a period Λ = 375 nm with a grating depth *d* = 36 nm, and included the presence of the 0th plasmonic order at the resonance wavelength excited by the counter-propagative − 1st diffracted order. The second simulated structure had a period Λ = 630 nm with a grating depth *d* = 54 nm, and included the presence of the − 1st propagative reflected order at the resonance wavelength excited by the + 1st evanescent diffracted order. The resulting dips were indeed around 635 nm, as shown in Fig. 1b. The plasmonic resonance of the first structure was deeper with a thinner FWHM than the plasmonic resonance of the second structure.

To identify the structure with the highest sensitivity, the growth of a layer of Al_2_O_3_ at the surface of the gratings was simulated. The thickness of the alumina varied from 0 to 15 nm every 1 nm and the resonance wavelength dip was calculated. Indeed in a range of alumina thickness of 20 nm, the resonance wavelength shift is considered as linear. Figure 1c plots the shifts in the wavelength of the plasmonic dip as a function of the oxide layer growth on aluminum. For the period Λ = 375 nm, the slope of the simulated curve is 0.499 nm/nm (alumina thickness) and for the period Λ = 630 nm, the slope of the simulated curve is 0.83 nm/nm. A greater slope indicates a greater wavelength shift, meaning that it will be more efficiently detected by the spectrophotometer during oxide growth, and may be linked to better sensitivity. The curve corresponding to the largest simulated period (Λ = 630 nm) had the highest slope in agreement with the literature where a greater period is said to enable better sensitivity^27^. The authors considered 630 nm for the optimized grating period but further optimization should have been done considering the resonance wavelength, angle of incidence to improve the sensitivity as well as the limit of detection. A much greater period would have made it possible to obtain even greater sensitivity, but would not enable a plasmonic dip at 635 nm with an incident angle of 20° in air. One has to note that a larger period will give rise to further propagative diffracted orders and thus will reduce the reflection of the 0th reflected order outside the resonance.

### Fabrication and characterization of the samples

**Figure 2:**
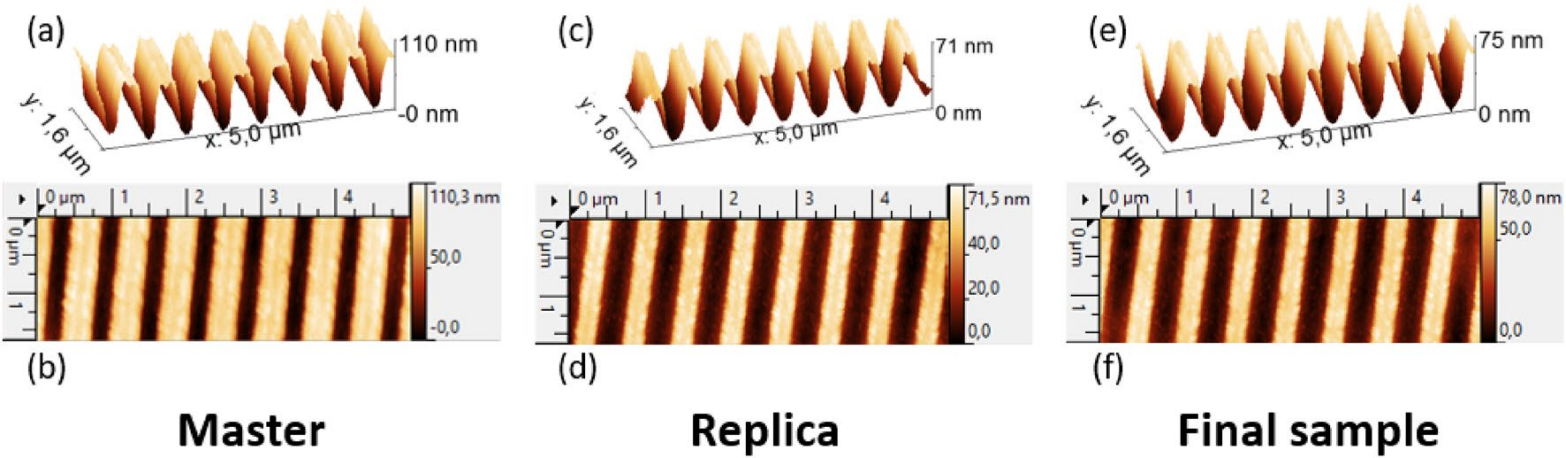
AFM profiles (**b**,**c**,**e**) and images (**b**,**d**,**f**) of master sample (**a**,**b**), Sol–Gel replica: (**c**,**d**) and final device with aluminum deposition: (**e**,**f**).

The first step was the fabrication of a master sample by interferential photolithography^28^ with a period Λ = 630 nm and a depth d = 80 nm. The AFM profile of this sample is shown in Fig. 2a,b. This grating is deeper than the simulated one due to grating depth reduction during the replication and annealing processes. But, after being replicated by nanoimprint lithography, the resulting replica had a period Λ = 630 nm and a depth d = 53 nm; the AFM profile is shown in Fig. 2c,d. With the replicated sample, roughness of the embossed sol–gel layer is reduced and measured in the range of 5–7 nm (Ra).

The sample was characterized once again using AFM measurement after aluminum deposition to identify the exact profile of the final samples. Figure 2e,f show that after aluminum deposition, the grating profile is almost the same as it was in the previous step. Finally, 150 nm thick Al layer was deposited using sputtering magnetron deposition. This thickness is far away from the penetration depth (skin depth) in the metal layer of the surface plasmonic mode propagating at the metal/air interface, which is around 30 nm for the visible wavelength range^29^.

**Figure 3:**
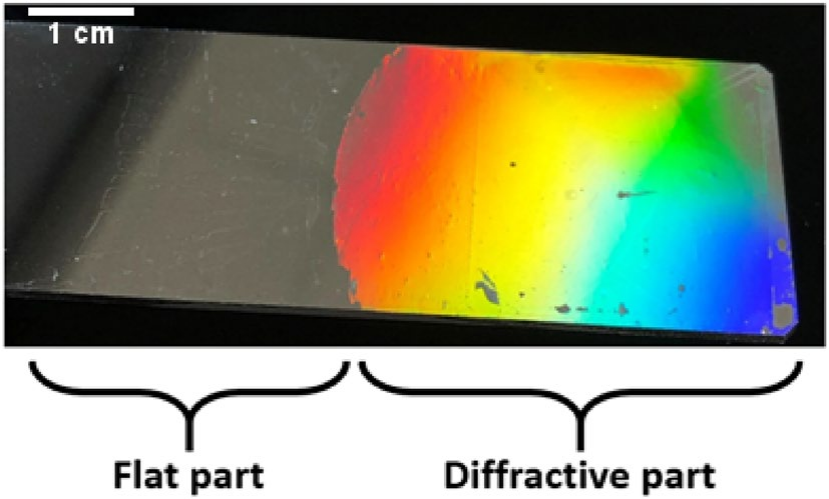
Picture of the final diffraction grating sample.

The final sample is shown in Fig. 3 illustrating the grating area (with the diffraction effect in the − 1st reflected diffracted order) and the flat area allowing the reference measurement.

### Experimental setup

**Figure 4:**
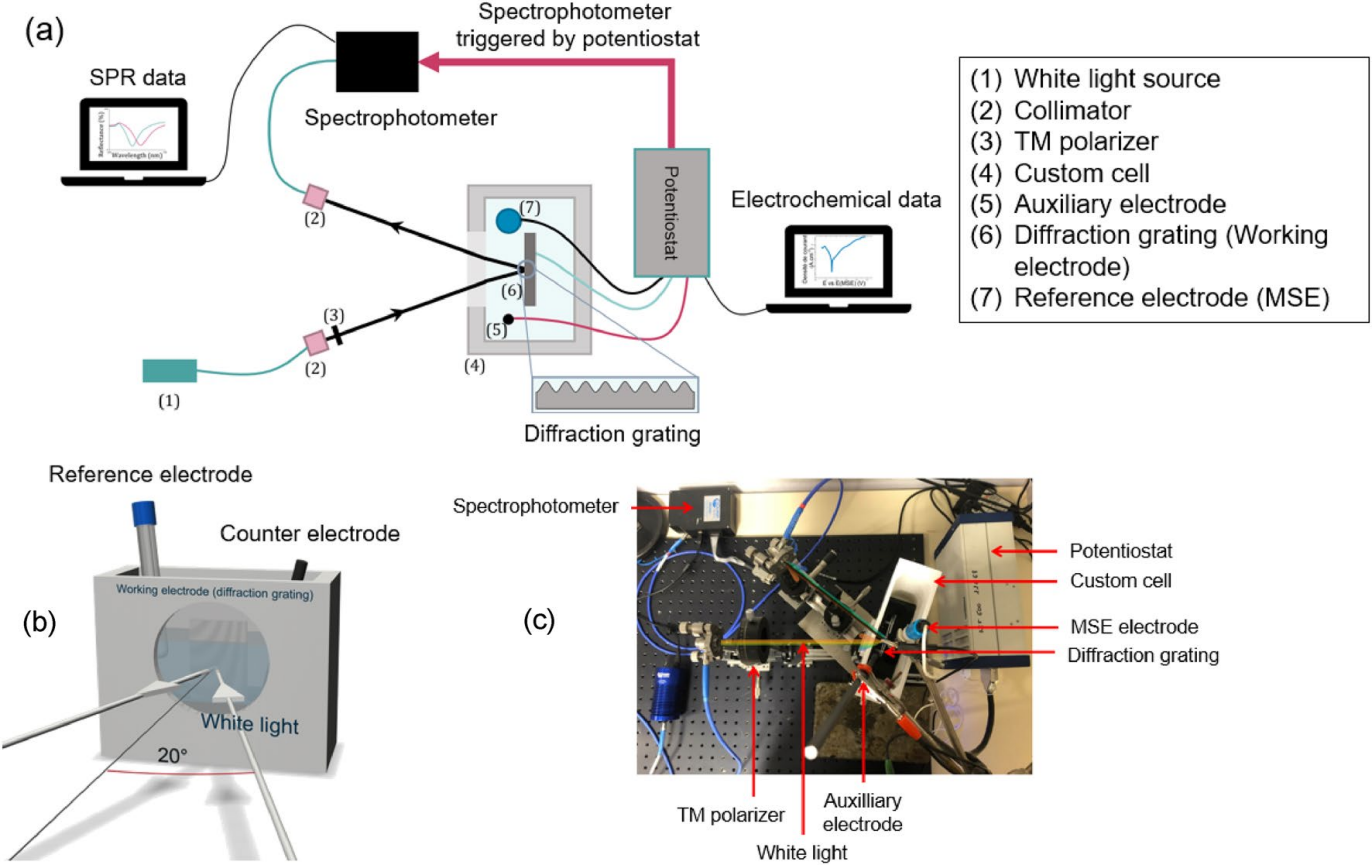
(**a**) Description of the complete opto-electrochemical set-up, (**b**) 3D schematic of the constructed cell allowing coupled optical and electrochemical measurements. (**c**) Picture of the experimental setup, with the incident TM polarized white light, the customed cell with the 3-electrode setup and the diffraction grating.

Next, the sample was characterized optically and electrochemically. To allow these coupled measurements as it is described in Fig. 4a, a cell had to be designed and implemented to perform spectroscopic and potentiostatic measurements. Figure 4b is the 3D schematic of the custom cell. The materials composing it should be inert to avoid influencing the electrochemical measurements. In these conditions, a nylon-based cell with an opening at the top was used to make the measurements and a removable Teflon sample holder was used to hold the diffraction grating. A sealed quartz glass was placed in front of the cell to enable the optical measurements. As mentioned above, the grating is used as a working electrode and is immersed with its lines oriented vertically in the cell filled with the electrolyte. A pincer on the sample ensuring the electrical contact. The reference electrode was placed to the left of the grating to ensure a stable reference potential and to make each measurement according to this potential. The counter electrode on the right closes the circuit, thereby enabling transport of the electric charges, and hence allowing the current to flow in the solution. These elements inside the tank comprised the 3-electrodes setup needed for the potentiostatic polarization measurements. Figure 4c is a top view of the entire setup. The path of the light beam is highlighted in the picture. The metallized grating is immersed in electrolyte and the flat aluminum part shown in Fig. 4 is used as spectroscopic reference. The potentiostat is connected to the electrodes (reference, auxiliary and the diffraction grating), which are partially immersed in the electrolyte. The third electrode (working electrode) is the grating sample.

### Evolution of the aluminum/electrolyte interface

**Figure 5:**
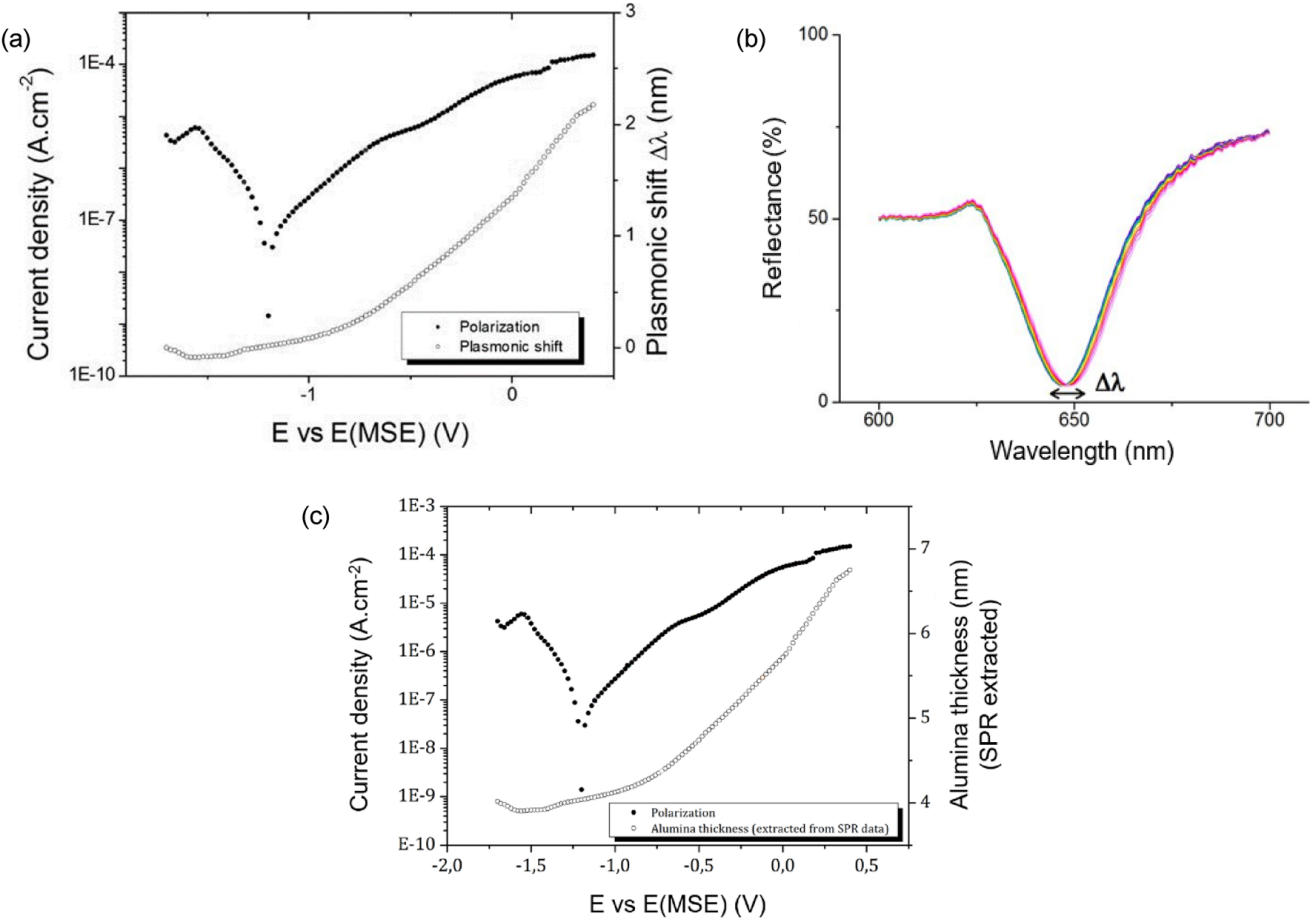
Polarization curve (black dots) and surface plasmon resonance wavelength shift (**a**) or alumina thickness extracted from the SPR (**c**) as a function of the voltage applied to the metallized diffraction grating after 1 h at OCP. (**b**) Reflectance of the plasmonic curves acquired at different potentials (− 1.7, 0 and 0.14 V) as a function of the wavelength.

Using this experimental setup, the aluminum/electrolyte interface of the diffractive sample can be monitored via SPR spectroscopic and electrochemical measurements. A potentiostatic polarization measurement is carried out by applying different potentials to the sample ranging from − 1.7 V to + 0.14 V versus E(MSE) with a 20-mV increase. The measured stabilized current density was averaged for each applied potential making it possible to obtain the potentiostatic polarization curve plotted in black dots in Fig. 5a. The polarization curve makes it possible to obtain further information about the electrochemical behavior of the interface aluminum/electrolyte, its cathodic and anodic domain and the measured current density as a function of the applied potentials. From − 1.7 V versus E(MSE) to − 1.2 V versus E(MSE), the curve corresponds to the measurement of reduction phenomena at the surface of the material. This is mainly the reduction of the H^+^ proton and dissolved oxygen. The current density reaches its minimum value at around − 1.2 V versus E(MSE), which corresponds to the corrosion potential in this environment. Beyond this, after the corrosion potential, the current density increases again and characterizes the anodic behavior of aluminum. The current densities are relatively low, indicating the presence of a passive Al_2_O_3_ film. The sample is dipped in a neutral pH electrolyte. In neutral aqueous medium, according to the Pourbaix diagram of aluminum^30^, the passive state exists between − 1.06 V versus E(MSE) and 0.14 V versus E(MSE). These are the potentials that were applied during the measurements and indeed suggest the presence of a passive film at the surface of the metallic sample.

Polarization curve (black dots) and the alumina thickness extracted from the SPR data (empty circles) as a function of the voltage applied to the metallized diffraction grating after 1 h at OCP are shown in Fig. 5a. Optical measurements (SPR spectra) were performed in parallel with electrochemical measurements. When the potentiostat began its acquisition, it sent a signal via the output signal to the spectrophotometer to start the acquiring spectrum (Fig. 5a). The experimental curves are slightly different from the simulated ones, due to the grating fabrication which leads to inhomogeneity and differences between the profile and the depth of the grating as well as grating roughness. Nevertheless the resonance wavelength shift is easy to measure and allows to monitor the oxide growth. The spectra were then processed to extract the shift in the wavelength resonance (“Supplementary Information”). To this end, all the spectra associated with an applied potential were averaged. Some averaged spectra are plotted in Fig. 5b for different applied potentials, where a wavelength shift Δλ in the plasmonic dip is visible. The Fig. 5b shows an enlargement of the dips to highlight the shift between the different spectra. The authors assumed that the process of the alumina growth was considered slow according to the response of the SPR. This means that the acquired spectra within 250 ms delay is highly sufficient to monitor the potensiostat measurements.

A negative shift in the plasmonic wavelength was observed during the first applied potentials. This small decrease corresponds to a change in the refractive index at the aluminum/electrolyte interface. This change may be explained by the reduction of the native oxide layer formed on the metallic sample. Next, a positive increase in the plasmonic shift occurred, before reaching the corrosion potential and continuing to increase until the end of the measurement. This positive shift can be linked to a variation at the electrochemical interface, namely the growth of a passive film, certainly combined with an evolution of the stoichiometry of the passive film and the double layer at the oxide/electrolyte interface. However, it has been shown that the double layer effect on the surface plasmon resonance is small compared with oxidation of the interface^31^. Oxide layers thicknesses have been retrieved from the SPR spectra using the modeling spectra and the known Al_2_O_3_ permittivity from the literature^26^. The simulated spectra allowed to directly determine the value of the respective alumina thickness according to the resonance wavelength shift. With the assumption of a homogenous permittivity of the passive film, we were able to calculate the alumina thickness during the electrochemical process, starting from 2 up to 7 nm, with an estimated uncertainty of 0.1 nm, considering the uncertainty on wavelength shift. Calculated thicknesses are plotted in Fig. 5c.

Similar trends were observed in the optical and electrochemical data, leading us to hypothesize that the surface plasmon resonance was indeed able to detect the aluminum passive film modification (stoichiometry, thickness, …). However, these measurements do not provide enough information to know the absolute thickness value and the characteristics of the passive film, i.e. its oxy/hydroxide combination, nor the characteristics of the electrochemical double layer and the electrochemical interface. These characteristics can be obtained by further measurements, such as Electrochemical Impedance Spectroscopy, which provide information on the physical properties of the oxide^32^. The thickness increases of the passive film can be estimated with this measurement and with the dielectric permittivity of the passive film, which would enable further research to correlate the detection of passive films with surface plasmon resonance.

## Conclusion

This study demonstrates that surface plasmon resonance (SPR) makes it possible to detect the growth of aluminia passive film during an in situ electrochemical process. The measured shift in wavelength of the SPR dip resonance showed a constant change at the metal dielectric interface during the electrochemical process. This negative shift could be linked to the reduction in the native passive film naturally formed on the sample before the measurements began. The positive plasmonic shift may be linked to the modification of the electrochemical interface and to the growth of a new oxide layer, thus an aluminum passive film. The resulting polarization curve as well as the knowledge concering the aluminum passive film enables us to consider the presence of a passive film between − 1.06 V versus E(MSE) and 0.14 V versus E(MSE). The optical and electrochemical measurements we carried out showing means we were indeed able to observe changes in an aluminum passive film through surface plasmons using a diffraction grating in a 0.02 M Na_2_SO_4_ electrolyte. With the assumption of a homogenous permittivity of the passive film, we were able to calculate the alumina thickness during the electrochemical process, starting from 2 up to 7 nm, with an estimated uncertainty of 0.1 nm, considering the uncertainty on wavelength shift. However, uncertainty remains concerning the exact composition of the electrochemical interface, the oxyhydroxide composition of the passive film, its thickness during growth, and the characteristics of the electrochemical double layer. Further studies focused on the coupled measurement, plus further electrochemical studies for an application in corrosion science, will advance our knowledge of these aluminum passive films through plasmonic measurements.

There is no direct application beyond the Al_2_O_3_ growth monitoring but these measurements are considered very useful for the electrochemist and thin passive film’s communities.

Future works will consist in investigating the flux and energy of the illumination. Indeed the authors would like to investigate the influence of performing optical SPR and electrochemistry measurements simultaneously and how SPR electrical field enhancement at the metal interface can influence the electrochemistry measurement, according to energy (resonance wavelength) and source fluence.

## Material and methods

The diffraction gratings used in this study were first simulated in water to determine their theorical plasmonic response before being fabricated using ultraviolet photolithography. The software used was McGrating^33^ based on Chandezon’s method^34^. The refractive indices of water, aluminum and Al_2_O_3_ used in the simulation were those implemented in the software. This simulation allowed to determine the design of the grating enabling surface plasmon excitation under white light illumination.

### Lithography process

A Neyco 2" Silicon wafer was cleaned for 10 min in an acetone ultrasonic bath and then placed in an ethanol ultrasonic bath for 10 min to remove any impurities. The wafer was then rinsed in deionized water and dried. A Shipley S1805 positive tone photoresist layer was then deposited homogeneously on the substrate by spin-coating at 3000 rpm for 10 s and then at 5000 rpm for 50 s. A Laser Interference Lithography (LIL) set-up with a 442 nm He-Cd laser of output power 200 mW, structured then the photoresist. The device consists of a beam splitter cube and a system for injecting light into two single-mode polarization-maintaining fibers at the laser wavelength. The two spherical waves coming from the optical fibers are then recombined on the sample surface. The principle is based on the interference phenomenon which creates constructive and destructive fringes whose period is fixed by the angle of the two overlapping beams and the laser wavelength. This setup made it possible to obtain periods ranging from 260 to 1310 nm. The article^34^ presents in more details the LIL procedure. The sample was then placed in an MF-319 developer for 3 s resulting in the nanostructured sample, thus creating the grating with a quasi-perfect sinusoidal profile. The authors considered a sinus profile for the modeling according to the AFM analysis of the fabricated gratings*.* The photoresist used to print the diffraction grating absorbs water easily, meaning diffraction grating exposed in aqueous media will be deformed by water absorption. To overcome this problem, we decided to replicate the diffraction grating by nanoimprint in Sol–Gel to have a mechanical and chemically stable grating in the electrochemical solution.

### Nanoimprint process

**Figure 6:**
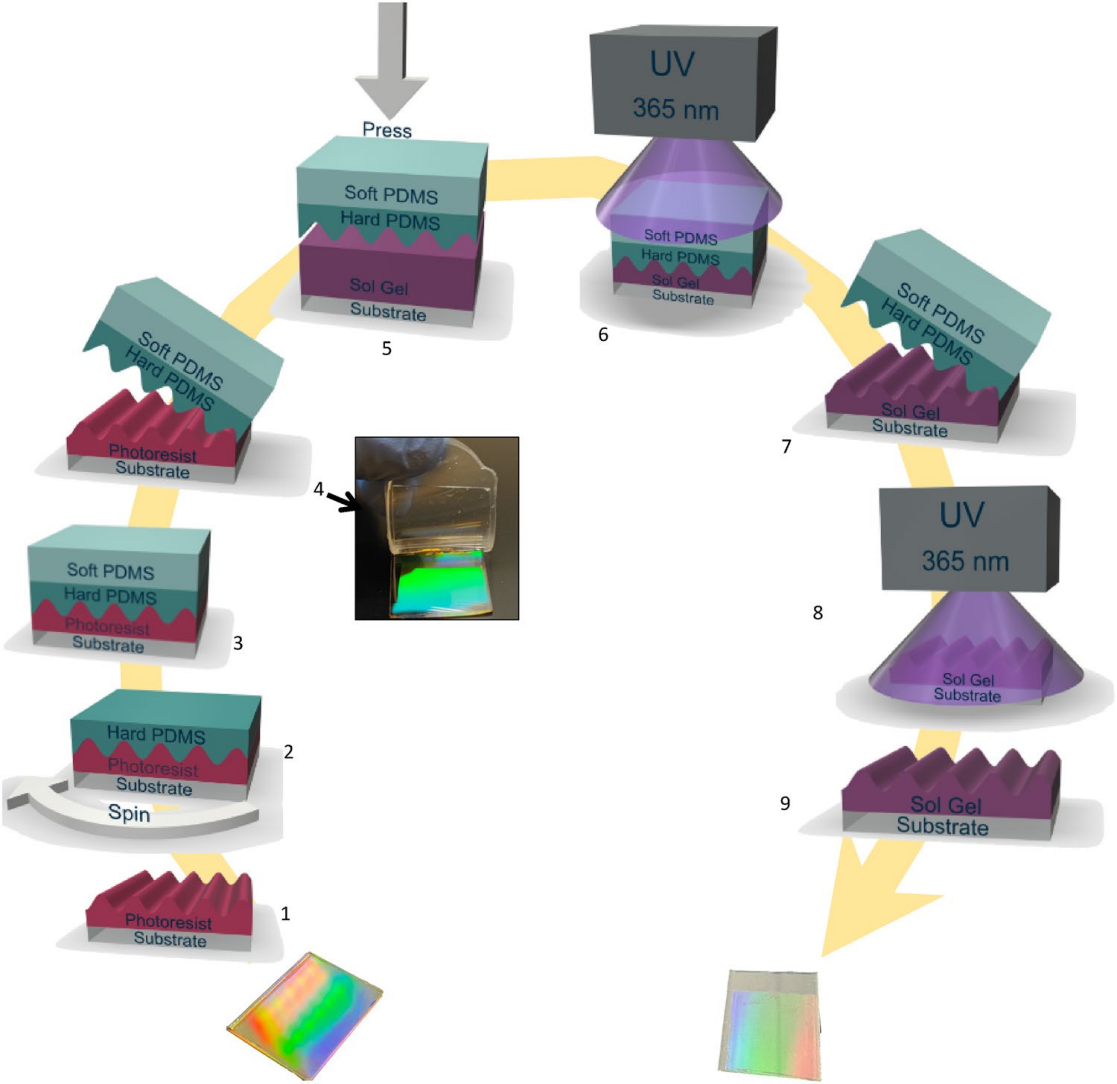
Schematic view of nanoimprint process.

The entire nanoimprint process^35^ was done by SILSEF Company. After realization of the masters by LIL (Fig. 6(1)), the PDMS stamps are made of two-layered PDMS (Fig. 6(2–4)). The first layer constituted of hard PDMS (Fig. 6(2)) is used for reproducing the accurate features of the master and for providing mechanical stability during molding. It is synthetized by mixing silicone and a crosslinker in a 2:1 weight ratio. After deposition, the sample is placed in an oven at 70 °C for 10 h. The second layer of soft PDMS (Fig. 6(3)) is used to provide flexible support for the hard PDMS and ensures pressure homogeneity during subsequent nanoimprint. It is synthetized by mixing silicone and a crosslinker in a 9:1 weight ratio. These layers are deposited by spin-coating at 3000 rpm for 10 s and at 5000 rpm during 50 s. Then, the sample is heated at 70 °C for 10 h. After, the stamps is removed from the master and the total thickness of the PDMS stamp is around 2 mm (Fig. 6(4)). Then, the PDMS stamp was placed in contact with the sample in a press, and a low-imprint pressure of 1 Bar was applied for 30 s (Fig. 6(5)). The sample was then illuminated with a UV lamp at 365 nm for 1 min at a power of 700 mW (Fig. 6(6)), to harden the imprint the SILSEF Sol–Gel formulation. Finally, the stamp was released from the replica (Fig. 6(7)), and the sample was illuminated with a UV lamp at 365 nm for 10 min at a power of 700 mW to stabilize the film (Fig. 6(8)).

The depth of the grating was adjusted based on annealing time (80 °C for a few minutes). This heat treatment slightly densifies the films without altering the lattice and period of the structures. The 150 nm aluminum layer was then deposited on the resulting replica by RF magnetron sputtering performed by HEF Company. Another motivation of replicating the diffraction grating using nanoimprint lithography is that it is expected to be possible to produce the final device at industrial scale. The entire NIL process is described in Fig. 6, from the photoresist-based grating fabricated by LIL to the copy of several gratings in sol–gel layer using the NIL process. Aluminum layer of 150 nm thickness was then deposited on the replicated gratings using Al sputtering process. Al layer thickness is not critical as long as it must be considered infinite by the plasmon mode, i.e. more than 30 nm which above the 1/e attenuation of the SPR mode electric field (evanescent field) in the metal layer, corresponding to the penetration depth in the metal.

### Characterization

The depth and profile of the gratings were characterized using an Atomic Force Microscope Bruker ICON while the grating period was determined by Littrow mounting measurement with a 635 nm laser diode. Finally, the aluminum sample/electrolyte interface was then characterized by spectroscopy and electrochemistry in a dedicated combined opto-electrochemical set-up in a transparent cell (tank), allowing both optical (SPR spectra) and current/voltage measurements during the electrochemical process.

The spectroscopic characterization was performed using an Ocean Optics halogen TM polarized white light HL-2000 that is incident on the aluminum diffractive sample fixed in the cell (filled by the electrolyte solution) with an incident angle of 20° outside the cell in air, through the transparent and hermetic window. The white light collimated by a reflective collimator (Thorlabs RC02FC-P01) passes through the TM polarizer, then goes to the custom cell to be incident on the diffraction grating, before being reflected for collection by the spectrophotometer. The size of the beam reaching the sample was around 2 mm diameter. The reflected beam is collected by a reflective collimator (Thorlabs RC02FC-P01) and observed with an Ocean Optics HR2000 + spectrophotometer using OceanView software.

The electrochemical measurements were performed with a Gamry Reference 600 that synchronized the acquisitions of the spectrophotometer, thus enabling synchronized signals to be acquired. The electrolyte was 0.02 M Na_2_SO_4_ (sodium sulfate) a pH neutral solution. A basic 3-electrodes setup was used. An OrigaLys saturated Mercury Sulfate Electrode (MSE) Hg/Hg_2_SO_4_, with a potential E =  + 660 mV versus the Standard Hydrogen Electrode (SHE) was used as a reference electrode. A graphite rod was used as auxiliary electrode and the metallized diffraction grating (sample) was used as the working electrode. The potentiostat measures the corresponding current density for each potential. These electrochemical measurements have to be made at the surface of the aluminum sample in steady state between the charges in the solution and the aluminum substrate. The working electrode was left in the electrolyte at Open Circuit Potential (OCP) for one hour to ensure steady state behavior. The potentiostatic polarization measurement began at the end of the OCP measurement. The potential was applied to the sample for two minutes, during which we allowed the current density to relax to record the stationary current density reached during the last 10 s of polarization. Only the data measured during the last 10 s were taken in account. When the potentiostat began its acquisition, it sent a signal via the output signal to the spectrophotometer to start the acquiring spectrum every 250 ms. The first forty spectra are then averaged to obtain a spectrum from which the wavelength resonance value is extracted. After data acquisition, measurement was paused for five seconds left before the potential was increased and a new step measured. When all the measurements were complete, the electrochemical data were processed.

The data collected during measurements were post-processed using MATLAB R2019a software.

### Supplementary Information

Supplementary Information.

## Data Availability

The datasets used and/or analysed during the current study available from the corresponding author on reasonable request.
