# Supplementary material for: In situ monitoring of thin alumina passive film growth by surface plasmon resonance (SPR) during an electrochemical process
Source: Sci Rep. 2024 Jun 14;14:13804. doi: 10.1038/s41598-024-64378-w (PMC11178765; doi:10.1038/s41598-024-64378-w)
Supplement: Supplementary file 1 — Supplementary Information. [file 41598_2024_64378_MOESM1_ESM.docx]

Supplementary information

In situ monitoring of thin alumina passive film growth by surface plasmon resonance (SPR) during an electrochemical process

J. Dutems^1^, N. Crespo-Monteiro^1^, F. Faverjon^2^, V. Gâté^3^, D. Turover^3^, S. Marcellin^4^, B. Ter-Ovanessian^4^, C. Héau^2^, I. Verrier^1^, B. Normand^4^, Y. Jourlin^1^*.

^1^Université Jean Monnet Saint-Etienne, CNRS, Institut d’Optique Graduate School, Laboratoire Hubert Curien UMR 5516, F-42023, SAINT-ETIENNE,

^2^HEF IREIS, 42160 Andrézieux-Bouthéon, France

^3^SILSEF, 74160 Archamps, France

^4^Univ Lyon, INSA Lyon, Université Claude Bernard Lyon 1, CNRS, MATEIS, UMR5510, 69621 Villeurbanne, France

*yves.jourlin@univ-st-etienne.fr


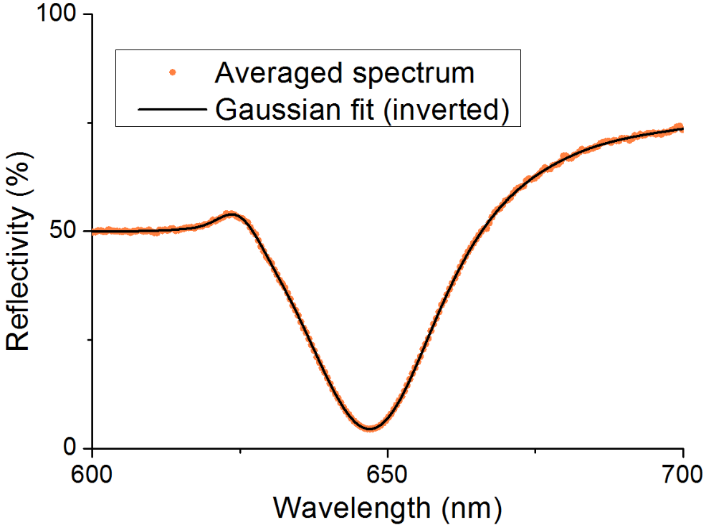


Figure S1. The orange curve represents the averaged spectra corresponding to the first potential, the black curve represents the corresponding 3^rd^ order Gaussian fit, inverted in this figure.

The spectra were inverted and fitted with a 3rd order Gaussian fit using the “fit” function in MATLAB to obtain a fit with 95% confidence bound. The averaged spectrum associated with the first applied potential is plotted in Figure S1 (orange dots) and the black line represents the corresponding inverted 3^rd^ order Gaussian fit. The resulting fitted curve corresponds to the measured data and confirms the relevance of using a 3rd order Gaussian fit in this case. The equation of the fit is then derived and the search for the zero derivative makes it possible to obtain the minimum of the fitted curve needed to extract the corresponding resonance wavelength for the spectrum associated with every applied potential with the greatest possible accuracy.
